# “The people who depended on us became expendable”: Experiences of frontline workers with lived and living expertise of drug use during the COVID-19 pandemic

**DOI:** 10.1186/s12954-025-01221-z

**Published:** 2025-05-18

**Authors:** Kat Gallant, Kelsey A. Speed, Alex Betsos, Brandi Abele, Matthew Bonn, Frank Crichlow, Alexandra de Kiewit, Michael Nurse, Alex Sherstobitoff, Natasha Touesnard, Karen Turner, the National Working Group of People with Lived and Living Expertise, Jade Boyd

**Affiliations:** 1 British Columbia Centre On Substance Use, 1045 Howe St, Vancouver, BC Canada; 2 Interdisciplinary Studies Graduate Program, University of British Columbia, 380-1961 East Mall Suite 380, Vancouver, BC Canada; 3 Canadian Research Initiative in Substance Matters, 1045 Howe St, Vancouver, BC Canada; 4 Department of Science and Technology Studies, Rensselaer Polytechnic Institute, 110 8th Street, Troy, NY USA; 5 Department of Medicine, British Columbia Centre On Substance Use, University of British Columbia, 317-2194 Health Sciences Mall, 1045 Howe St, Vancouver, BC V6Z2A9 Canada

**Keywords:** Frontline, COVID-19, People who use drugs, Overdose, Harm reduction

## Abstract

**Background:**

This paper explores the perspectives of a group of people with lived and living expertise of unregulated drug use who worked as frontline harm reduction service providers and activists in Canada during the dual public health emergencies of COVID-19 and the toxic drug crisis. Specifically, this paper explores their initial experiences at the onset of the COVID-19 pandemic, their reflections on how these experiences varied one year into the pandemic, and their perspectives on how shifting public health measures and policies shaped their work.

**Methods:**

Drawing on collaborative research methods, this project was developed with a national working group of people with lived and living expertise of substance use. Three rounds of roundtable discussions along with two sets of semi-structured interviews were conducted with working group members from May 2020 to June 2021. A thematic analysis was co-developed by academic facilitators and the working group through deductive and indictive group coding and reflexive analysis.

**Results:**

Four themes emerged from the interviews and roundtable sessions: (1) initial negative impacts of COVID-19-related public health measures; (2) societal abandonment, collective anger and grief; (3) navigating constantly shifting public health emergencies over time; and (4) leveraging lived expertise to create positive change.

**Conclusions:**

The COVID-19 pandemic, in concert with the toxic drug crisis, presented many challenges for working group members on the frontlines to continue their work providing essential services to people who use unregulated drugs in Canada. The experiences shared by this unique group demonstrate these challenges, as well as how the immediate and long-term impacts of these dual public health emergencies provided opportunities for innovating and advocating for sustainable policy changes.

## Background

The unregulated drug supply in Canada has led to a nationwide crisis of overdose deaths. Between January 2016 and March 2024, there were 47,162 apparent opioid-toxicity deaths reported across Canada, and nearly 70% of these deaths occurred between January 2020 and March 2024^1^ [63]. The exacerbation of the drug overdose crisis during the COVID-19 pandemic has largely been attributed to disconnected public health policies designed to reduce disease transmission, which failed to account for the unique needs of people who use unregulated drugs, and the increasingly volatile unregulated drug supply (e.g., fentanyl and its related analogues) [12]. At the beginning of the pandemic, public health guidelines focused on physical distancing measures. These measures resulted in closures and/or reduced hours of operation of harm reduction and other essential services, as well as an increase in people using unregulated drugs in isolation (which is associated with an increase in overdose risk) [20, 30, 54]. Border closures led to supply chain issues which contributed to disrupted access to unregulated drugs and a dramatic rise in prices, as well as increased toxicity and impurities of the unregulated supply (e.g., with contaminants and adulterants such as benzodiazepines) [1, 30]. In response, many jurisdictions across Canada made changes in prescribing policies to reduce the use of the poisoned unregulated drug supply (e.g., expanding the eligibility for prescribing take-home opioid agonist treatments) [47, 66]. Provinces and territories eventually reopened some harm reduction services (albeit with restricted hours of operation) [21], and several municipalities implemented initiatives to reduce the spread of COVID-19 in unsheltered populations by providing temporary housing (e.g., temporary ‘pop-up’ shelters and hotel rooms) to promote isolation [3, 25, 32], particularly for individuals who contracted the virus. Despite these advancements, the crisis continued to worsen with a 32.9% increase in overdose-related deaths nationwide in 2021 compared to 2020 [70].

People with lived and living experience and expertise of unregulated drug use working in harm reduction services (referred to herein as ‘workers with lived expertise’^2^) are an essential component of the response to the toxic drug crisis. As workers with lived expertise have shared understandings of the specific needs and challenges experienced by people who use unregulated drugs, they are uniquely positioned to respond to community needs compared to workers without lived experience and/or external to the communities they serve [5, 36, 59]. Workers with lived expertise act as a bridge between people who use unregulated drugs and harm reduction services, serving as trustworthy and nonjudgmental resources with experiences navigating community-based drug use-related services, ultimately making services more accessible and effective [5, 36, 59]. Organizations led by people with lived expertise have always been at the forefront of harm reduction efforts in Canada, evidenced by decades of political activism and implementation of innovative solutions for their communities. These organizations and allies have opened (necessary) unsanctioned peer-driven overdose prevention sites (a designated space where people can use drugs under the supervision of peers who are trained to respond to overdoses) [17, 45], syringe distribution programs [38], and, most recently, an unsanctioned compassion club that distributes tested unregulated drugs (for known drug content and purity) to those at high risk of overdose (i.e., regularly using unregulated drugs) in Vancouver, British Columbia [43].

Despite the benefits of prioritizing workers with lived expertise as harm reduction workers, literature has demonstrated that this work can result in detrimental impacts to their well-being, particularly in the absence of institutional and social supports [56, 57]. Burnout is a major concern among workers with lived expertise, largely stemming from repeated exposure to preventable death and overdoses [50], the demanding nature of this work in the context of criminalization, and their systematically underpaid and undervalued labour [35, 50, 76]. As the COVID-19 pandemic was associated with experiences of burnout for the workforce more broadly, there is a lack of literature examining the experiences of workers with lived expertise on the frontline of the toxic drug crisis. This paper draws from research examining frontline workers’ experiences during the COVID-19 pandemic, including how shifting public health measures and policies impacted their efforts to address the toxic drug crisis in Canada. Specifically, this paper reviews their initial experiences providing harm reduction care at the onset of the COVID-19 pandemic, how their experiences changed over the first year of the pandemic, and the innovative and pragmatic ways they continued to provide care and support to people who use unregulated drugs throughout the dual public health emergencies.

## Methods

### The working group

This research originates from the People with Lived and Living Expertise of Drug Use National Working Group (referred to herein as the ‘working group’), a component of the Canadian research consortium, CRISM [16]. The working group has been described in detail in other works [7, 62], and is further summarized here. Established in 2018, the working group brings together people with lived and living experience in substance use with expertise in frontline service provision in harm reduction who act, not just as research participants, but as co-investigators who shape how projects are instigated, designed and implemented in collaboration with academic researchers. Working group members are experts in the field of substance use, representing national and regional drug user unions including higher-level leadership and founding members. Members have effected change on both the local and international stage, having been consulted with by all levels of the government and local health authorities, as well as the United Nations, on drug-related policy and programming issues. They have also acted as investigators on research studies, authored numerous publications in peer-reviewed journals on drug-related topics, and presented at conferences across Canada and internationally.

From January 2018 to December 2023, working group members and academic facilitators met remotely via telephone and Zoom at least monthly, with rotating working group member facilitation, to discuss and create actionable research addressing Canada’s toxic drug crisis. The working group facilitator worked alongside an academic facilitator to set the meeting agenda and lead the discussions during the meeting (with limited support from the academic facilitator, as needed). The meeting facilitator disseminated the meeting agenda prior to the monthly meeting, and an academic facilitator recorded and then shared meeting notes to the working group members. Working group members came from urban and rural settings and are associated with CRISM’s five regional nodes across Canada: British Columbia; Ontario; Quebec; Atlantic regions; and the Prairies [61]. Over the course of the initiative, we lost 4 of the 14 founding members. We grieve their loss and remember their spirit and dedication to creating change, and we carry their passion and learnings with us. Working group membership varied between 13 and 16 members at any given time, with active recruitment by working group members in collaboration with academic facilitators when needed to maintain that level of participation. Four academic facilitators supported the project, though not all at the same time, with the exception of JB who was present throughout.

The working group engages many principles of community-based participatory research (CBPR), which emphasizes community priorities, reciprocal knowledge transfer, shared decision-making power, and mutually beneficial outcomes between academic researchers and working group members [8, 41, 42, 65]. This methodological approach moves away from the traditional concept of academic research that is imposed “on” communities, towards a more equitable approach where research is conducted “with” communities. While the academic facilitators provided capacity-building support (e.g., furthering research skills through academic writing and reviewing, data analysis and interpretation, knowledge dissemination, and national network expansion) to working group members [7, 60, 61], the academic facilitators benefitted in equal measure from the working group members’ expert knowledge, and, as such, working group members led the conceptualization and development of this manuscript’s research topic, including study design; acted as participants by sharing their lived experiences through semi-structured interviews and group discussions; and supported the writing and reviewing of paper drafts (all of which included financial renumeration for their expertise). Overall, the academic facilitators acted as a bridge between academic institutions and the working group members, supporting the research process, including dissemination of research findings through jointly developed academic articles, conference presentations and other knowledge transfer materials (e.g., websites, photovoice journals, etc.). This methodological approach follows principles developed by other drug user groups (to empower research subjects’ autonomy) working collaboratively on research for social justice (see [9, 23, 48, 74]). This particular study was developed at the outset of the COVID-19 pandemic in response to collective group interest in documenting the challenges faced by working group members in their roles as frontline harm reduction workers and activists.

### Data collection and analysis

Between May 2020 and June 2021, two academic facilitators (AB, JB) conducted 15 interviews (9 initial interviews and 6 follow-up interviews) and 3 roundtable discussions with a total of 13 working group members participating via telephone or video call. Roundtable discussions were methodologically useful for sharing and building upon each other’s experiences and identifying salient collective themes [4], whereas individual interviews allowed for more nuanced, in-depth, personal discussion of experiences [22]. All working group members were invited to participate in interviews and roundtable discussions. While no working group members declined to participate, due to extenuating circumstances and life stressors, not everyone was able to participate in all activities within the given time periods. The project was completed in three stages, in order to follow the shifting landscape for frontline workers during the pandemic, with each stage generating the questions asked in following stages. After each stage, the interview questions were added and/or adapted based on topics discussed using an iterative process. Given the temporal nature of this study, a reflexive approach ensured that the questions remained relevant within the rapidly shifting landscape of the COVID-19 pandemic. For example, interview and roundtable questions early in the COVID-19 pandemic focused on immediate changes to the working group members’ frontline work, any observed and/or experienced new or enhanced barriers to accessing services and current community needs identification. Later interviews and roundtables, however, centred on new and ongoing challenges and measures being taken to address the dual pandemics and the lasting impacts on both working group members personally and as workers, as well as on the communities they serve.

The initial stage (“Stage 1”) of this study took place toward the end of the first wave of the pandemic (March—September 2020), as substance use related services began reopening from the Canada-wide lockdowns associated with the onset of the pandemic [14, 26]. Stage 1 included nine one-hour interviews (May 14—June 30, 2020) and one two-hour roundtable discussion (July 14, 2020, n = 8), which focused on the initial impacts of the pandemic. The second stage (“Stage 2”) of this study took place after the Canadian government announced the second wave of the pandemic (September 2020—March 2021) [69]. Stage 2 consisted of a two-hour group roundtable (October 13, 2020, n = 7) to discuss how the ongoing impacts of the pandemic, and new policy changes, were influencing their work and day-to-day lives. The third and final stage of this study (“Stage 3”) was conducted at the beginning of the third wave of the pandemic in Canada (March 2021—September 2021) [69]. Stage 3 consisted of six one-hour follow-up interviews (March 23, 2021—June 4, 2021) and a group roundtable (June 8, 2021, n = 8), and focused on the continuing and potential long-term impacts of the pandemic. All working group members provided verbal informed consent before each data collection session and received $30 CAD (provided via e-transfer or cheque) per hour as participants. This study received ethics approval from the Providence Health Care–University of British Columbia Research Ethics Board.

Transcripts of the interviews and roundtable discussions were de-identified and imported into NVivo 12 by the academic facilitators, and then primary themes (shared experiences) were shared with the working group during regular meetings to co-develop a code book to support thematic analysis across the datasets [19]. Initial themes emerged through deductive (e.g., worker experiences during the COVID-19 pandemic) and inductive (e.g., grief and anger, impact of shifting public health measures) group coding over several months [22]. More specifically, to assist the collaborative research process, identified themes, analysis, and findings were read out loud, discussed and continually reviewed by working group members at monthly meetings facilitated by rotating group members. In on-going conversation with working group members, themes were later refined for this manuscript to the four overarching themes discussed in the Results section. On-going monthly feedback, reflection and comments by the working group were incorporated by the academic facilitators until an approved draft paper was complete. The back-and-forth process with the working group was complex and sometimes messy (with sometimes divergent opinions) but also enabled a more collaborative framework and outcome prioritized by the group [9, 71]. While this paper was initially drafted by academic facilitators involved with the project, the working group reviewed, added to, and further edited drafts of this paper, and provided final approval for journal submission.

## Results

In total, 13 working group members participated in this study. Over half of the members identified as women (n = 7; 54%), and 6 identified as men (46%). The working group had some ethnic diversity, with Black, African-Caribbean and predominantly white members during this study period, however, we recognize these voices are not representative of all people with lived and living experience in Canada. While Indigenous members were part of the working group, they were unable to participate in this particular study, resulting in a distinct lack of Indigenous voices in the working group during the study period. The age range of working group members spanned over 30 years, with the youngest in their 20s. While members varied in their socioeconomic status throughout their lifespan and within the study period, it can be said that all have faced economic marginalization at some point in their life.

The working group members represent the following provinces across Canada: Ontario (n = 4, 31%), British Columbia (n = 4, 31%), Alberta (n = 2, 15%), Quebec, (n = 1, 8%) and Nova Scotia (n = 2, 15%). In individual interviews, working group members described their day-to-day frontline work prior to the pandemic as well as their continued work where possible. These responsibilities included outreach work (e.g., taking clients to appointments, recovery meetings and other addiction counselling services; securing and delivering harm reduction supplies; engaging with community members, assessing their needs, making referrals, and doing follow-ups; case management and client advocacy; and providing individual counselling to clients), while others worked as “peer support workers” in supervised consumption sites, and as harm reduction and public health educators (e.g., providing naloxone training or information on safe supply). While many working group members’ roles were altered by the pandemic, many of these responsibilities remained the same but sometimes in a different capacity as newer delivery models were implemented (e.g., virtual service provision).

Conducting the study over three temporal stages allowed us to better capture shifting experiences throughout the pandemic, from the initial months when harm reduction services were abruptly closed, to one year in when (many) services were reopened, but were drastically changed. Our analysis identified four major themes regarding the impact of the COVID-19 pandemic on working group members as workers with lived expertise, which emerged during—and at times extended between—stages. Themes that emerged in the first stage (representing the beginning of the pandemic) included “initial negative impacts of COVID-19-related public health measures,” including expressions of “societal abandonment, collective anger and grief.” Throughout the second and third stage, workers described the trials of “navigating the constantly shifting public health emergencies over time”. Forms of worker resilience were identified throughout each stage and are represented in the final theme—“leveraging lived expertise to create positive change.”

### Stage 1: May—July 2020

#### Initial negative impacts of COVID-19-related public health measures: *“You can’t just drop [people] and walk away”*

In the early days of the pandemic, many harm reduction services were closed or remained open at significantly reduced capacity in response to federal and provincial public health measures designed to reduce COVID-19 transmission. These service closures impacted the physical health of people who use unregulated drugs, but also the social and emotional connection they received through interactions with workers with lived expertise. One group member framed these service closures as disrupting workers with lived expertise’s “connection to hope”:

*One of the things that I believe outreach workers [with lived expertise] provide is a connection to hope. It is a connection to an awareness that as a user, as a person going through difficulties, you’re still a part of humanity. That somebody still cares. And the way that I see that is to be there and to be with people and to be at that level and that is something that people with lived experience can deliver because they can say ‘I know what this felt like. I know what it was like for me’.* (P3, May 2020, Interview)

Service closures and disruptions also led to job losses or significant changes in roles for working group members who were employed by these services (e.g., overdose prevention sites), which greatly impacted their own well-being. Many working group members explained their work brought them joy and personal fulfillment, through helping others and connecting with people in their communities. Their resulting loss of work contributed to their own loss of purpose, sometimes with dire personal consequences (e.g., increased intensity of substance use and overdose):

*I get a lot of joy out of [outreach] and I think that’s a lot of the reason why I crashed so hard too because I wasn’t able to do it [while] being locked down [due to pandemic policy-related restrictions] for so long, you know. I get a lot of joy out of just bringing a little bit of joy to somebody else.* (P2, May 2020, Interview)

When organizations began to provide in-person services again, public health measures such as physical distancing from others and wearing personal protective equipment (PPE) had a negative impact on their ability to build and maintain relationships with people in their community. A group member engaged in street outreach work described the limitations of required physical barriers when supporting community members: “You can only talk to one [person at a time]. You know, like you have to be distant, you got to wear a mask, and a shield, and all that. It’s like, that in itself is limiting.” (P9, June 2020, Interview). Other group members described it as feeling “like an imposter,” and that it “just didn’t feel right”; that the measures produced both an emotional and physical barrier that hindered “intimacy” when building relationships.

During this time, many services transitioned from in-person to online or telephone formats, which put a further strain on working group members due to lack of appropriate infrastructure and familiarity with the technology and new processes. It also contributed to the sense of being unable to connect with those they provided services to on a personal level—a central component of this work. As such, this shift from in-person to virtual work generated significant emotional distress for many group members, who expressed feeling guilty about not seeing community members face-to-face, stating that it felt like they were “abandoning” the people who depended on them for services (July 2020, Roundtable). Group members described feeling especially worried about being unable to provide appropriate support for community members who were particularly vulnerable to service disconnection:

*Sometimes I get a little antsy working from home. I don’t feel like I’m doing my part for the people who use drugs, you know. Because of my age and my experience, I know the much older clientele that some of our newer staff don’t know and nor does that population really want to get to know the younger population. So that clientele of mine is starting to really die out.* (P6, June 2020, Interview)

For some working group members, the pandemic posed a significant threat to their health due to their age and/or medical comorbidities that increased their risk of severe outcomes associated with infection [77]. As a result, they had to continue working from home, even as in-person services began to reopen, to protect themselves from COVID-19 exposure. Some described how this heightened their feelings of guilt for not being in person, stating that there “isn’t a whole lot you can do from home” (P6, June 2020, Interview), and describing the ability to stay safe at home as a “privilege”:

*It breaks my heart every time I open up an email and read that we lost another community [member] to an overdose. Then you feel guilty that you’re not out there, at least I feel guilty because I’m at home being safe. I feel guilty I’m not out there helping and I want to be but I’m not allowed to be.* (P6, June 2020, Interview)

For working group members, the uncertainties and frequently changing guidelines imposed upon harm reduction services materialized into concerns about the consistency of operating hours, capacity, and availability of these essential lifesaving services. One group member expressed their frustration with the rollback of critical services, arguing that “you can’t just drop [people] and walk away” (P3, May 2020, Interview). The theme of uncertainty was echoed throughout the pandemic by many in the working group, who witnessed significant upheaval of government policies impacting people who use unregulated drugs, both for the better and worse. For example, some safer opioid alternatives to the poisoned drug supply (e.g., take-home hydromorphone) were offered during the pandemic to support social distancing [58]. However, many group members predicted that this resource would be discontinued after the pandemic and feared that this would increase the risk of overdose among those currently accessing this safer supply if they were later only able to access adulterated street drugs. One working group member described the doubt people were having regarding the longevity of the safer supply program, which led to hesitation and fear to access the program:

*The fear is the post-COVID. [For example] what’s gonna happen when they drop the safe supply? That’s another thing people are in fear of. So I go in and I get a safe supply, they’re gonna give me a pile of dillies [Dilaudid] so I get used to taking them and then, all of a sudden, I got to go back on methadone, right? That’s a huge fear, and it’s holding people back from even accessing safe supply. It’s a big trust issue.* (P9, June 2020, Interview)

This feeling of uncertainty and frustration was compounded by reports that housing initiatives implemented early in the pandemic (e.g., temporary housing in hotels and shelters in certain municipalities in order to promote isolation and reduce the spread of COVID-19) were already being cut back. The inconsistent roll-out and removal of these interventions contributed to community members’ frustration at what they characterized as paternalistic government actions, which were perceived by group members to provide support only when people who use unregulated drugs were viewed as a risk to others:

*Well, we’re dealing with a new problem now, that everybody gets housed. Because they put everybody in shelters or the hotels, right? […] Now we’re finding out that all of that is ending at the end of this month and everybody’s just going to be put back out on the street. So, I mean, to me like it’s very… I mean, since when is it only important to house people when they pose a risk to our larger society? Like why is it that we don’t see that as a necessity to house people to begin with?* (P7, June 2020, Interview)

#### Societal abandonment, collective anger and grief: *“[T]he whole community is sort of suffering together”*

In the early months of the COVID-19 pandemic, working group members observed an insufficient focus on supporting people who use unregulated drugs in navigating the dual pandemics, which they viewed as largely responsible for the notable increase in both fatal and nonfatal overdoses. Working group members in this study were directly impacted by the devastating levels of death, as their friends, families, and communities were disproportionately burdened by this increase in overdoses. Many perceived this lack of support as abandonment by all levels of government (municipal, provincial, and federal), public health officials and the general public. Beyond the impact on their social and work lives, group members reported experiencing a deep sense of disappointment at the perceived regression in drug policy after fighting for years—sometimes decades—to reduce overdose-related deaths and harms. As one participant described:

*Well, to be completely honest, I’ve been down in the [neighbourhood with an open drug scene] for about 35 years, I guess. Maybe even a little bit more. But I don’t think I’ve ever seen quite how bad it is and some really dire consequences that are loading up that it’s going to be just a horrible situation. You know, the numbers as far as overdose resulting in death, they’re as high as they’ve ever been.* (P5, June 2020, Interview)

Working group members noted that the apparent prioritisation in public health messaging and practice of reducing COVID-19 transmission seemed to outweigh the risks associated with the toxic drug crisis, making people who use unregulated drugs feel “expendable” (P3, May 2020, Interview). In a roundtable discussion, one group member described how government policy ultimately prioritized COVID-19 public health measures over the safety of people who use unregulated drugs, providing “mixed messaging” that had lethal consequences:

*You know we had the mixed message where, you know, we want people not to shoot [drugs] alone but if you had COVID we wanted you to stay alone. So, there was all this mixed messaging. I can’t say that caused [the rapid rise in overdoses] or shutting down the OPS’s or limiting their hours was the cause. But cumulatively it just killed us.* (P4, July 2020, Roundtable)

The irony of the government and public health officials proclaiming that COVID-19 public health measures (that resulted in service disruptions) were in an effort to ‘protect the most vulnerable in society’ reinforced feelings of anger and a sense of expendability among group members dealing with overdose ‘vulnerability.’ Many group members described how the surge in overdose deaths had led to widespread experiences of grief and emotional harm among their communities. Members explained that community members all knew people who had died due to overdose and that their communities were getting smaller by the day, exemplified in one member’s recounting of their “shrinking” world:

*[The] world that I was a part of and still am a part of, but that world that held my story, certain stories that aren’t held out prior to that experience, that that world is shrinking. So my world is shrinking. The people that I could feel connected with as I walk the street, that is shrinking.* (P3, May 2020, Interview)

Others noted the tone among their social networks and their neighbourhoods had changed, as the impacts of collective suffering and extensive loss emerged. One group member described their community as experiencing cumulative post traumatic stress disorder (PTSD):

*But there’s this cumulative PTSD that the whole community is sort of suffering together in. You know what I mean? I’m trying to… it’s like a group of people that all have this stress disorder and the whole little [neighbourhood] has this stress. It’s not just an individual case of PTSD. It’s like the whole goddamn city has it—and it’s sort of all integrated and that is making for a really, really hard situation.* (P5, June 2020, Interview)

Their suffering was exacerbated by what they perceived as an undervaluing of overdose-related deaths in the media. Working group members described their frustration with double standards in the media that contributed to unbalanced reporting of COVID-related deaths alongside the continued lack of recognition and devaluing of the toxic drug crisis:

*[COVID is] all over the fucking news. Everything. We can have seven deaths in one week and not a fucking word on the news about the overdose and I believe, really believe that the overdose crisis is huger than the COVID. Still is. You know? And it seems that people have forgotten that unfortunately.* (P6, June 2020, Interview)

For group members, the frequent experiences of loss among their communities were further complicated by their inability to “say goodbye properly,” (P5, July 2020, Roundtable) to friends and community members given the physical distancing directives in place in the first few months of the COVID-19 pandemic and the restrictions on public memorials. Prior to the COVID-19 pandemic, group members were able to grieve and lean on others—particularly colleagues – which they described as essential for mitigating the burnout experienced by frontline workers, who largely lacked access to institutional grief and trauma support (e.g., paid time off, benefits, counselling) [7].

However, even when group members were allowed to see their colleagues in person, they noted their capacity to support each other during the COVID-19 pandemic was significantly reduced due to their emotional exhaustion, and the lack of access to mental health and wellness services and vacation time. One group member described how mutual informal support was disrupted during the pandemic:

*We, as workers, we support and look after the people who we take care of, our service users. But then we, as workers, who do we get support from? We don’t get support from anybody other than maybe our coworkers [and only] if they’re not too burnt out at the end of the day to even sit down and talk and say, you know, today was a rough day. I know you had a rough day because we had so many overdoses. You know, maybe, you know, ‘Is there anything I can do to help you?’ My co-worker might be too tired to even ask that question*. (P1, May 2020, Interview)

In summary, working group members emphasized how the early public health measures implemented in response to the COVID-19 pandemic caused harm due to service disruptions and the corresponding loss of personal fulfillment and moral distress in providing inconsistent care to people who use unregulated drugs. Group members also described the collective feeling of societal abandonment and substantive grief given the rising overdose death rates, as well as the ways in which they were expected to continue providing care, without the provision of institutional supports.

### Stages 2 & 3 [October 2020—July 2021]

#### Navigating the constantly shifting public health emergencies over time: *“The pandemic has done a lot of damage to us, … physically, mentally, and emotionally”*

Seven months into the COVID-19 pandemic (and beyond), the negative impacts of physical distancing and significant experiences of grief and overdose-death-related trauma continued to be relevant among group members. While many services began to reopen during this period, members recounted the sustained challenges in understanding which supports were available, and who could or could not receive them. Before the pandemic, community members learned about services through word-of-mouth and outreach workers, facilitated through the close ties between workers with lived expertise and their community. Working group members recounted witnessing and experiencing the frustration that came with navigating services and attempting to provide up-to-date information to those who needed it during this period:

*Are we actually getting the information to the people who need it? Probably not in the COVID era, right? Because a lot of people that we work with don’t have access to Internet or a computer or whatnot, so they may not have even seen these, you know, infographics that we’ve done.* (P7, October 2020, Roundtable)

The working group noted that the dwindling of community networks due to communication challenges were further exacerbated by “haphazard” service disruptions as well as through “death,” and “displacement.”

The ongoing navigation of the dual public health emergencies took its toll on frontline workers. Exhaustion, personally and professionally, contributed to their mounting burnout. As one working group member described: “The pandemic has done a lot of damage to us, and that’s what we are dealing with – I am dealing with right now, physically, mentally, and emotionally” (P1, April 2021, Interview). This exhaustion significantly impacted group members’ reactions to learning about recent deaths in their communities; reports of feeling ‘numb’ when learning about new losses were particularly concerning among group members. They described experiencing fewer ‘human emotions,’ with one member reporting that they no longer cried after learning about deaths: “you get used to it, and that’s terrible. How can you get used to something like that?” (P13, June 2021, Interview). This dissociation from grief was noted as a significant ongoing, long-lasting impact of the dual public-health emergencies that workers with lived expertise were both victims of, and witnesses to, given their positions as both community members and frontline workers:

*And then of course there’s the deaths. I was talking to somebody the other day and they told me man, ‘I know more dead people than I know living people’. Like pretty much all the people that held your story, that could affirm you are pretty much gone. And it’s this numbness to death and to the absence of people in your life.* (P3, May 2021, Interview)

Navigating new, continually shifting landscapes of service provision, alongside dramatic increases in overdose deaths over-time and resultant disassociations from grief, culminated in feelings of demoralization among group members.

### Stages 1, 2 & 3 [May 2020 – June 2021]

#### Leveraging lived expertise to create positive change: *“We have to make a better place, you know? And hopefully we’ll do it”*

While the COVID-19 pandemic presented challenges to all aspects of working group members’ lives, it also inspired short-term strategies to meet immediate community member needs and long-term advocacy goals to push for sustainable policy change. Members described the crucial role of information access, and the vital act of sharing information as a form of resilience in the face of structural barriers. Workers with lived expertise continued to reach out to and support community members via various innovative knowledge sharing strategies, prioritizing the preparation and sharing of posters and information packages, which allow community members to access information and resources on their own time. Others supported various virtual adaptations, including implementing informal and formal video calls for witnessed injections to accommodate those who cannot access supervised consumption sites, and providing short-term prepaid phones for people who use unregulated drugs to connect directly with frontline workers who could then continue doing wellness checks and provide necessary supplies (e.g., groceries and medications). Others described organizing individuals to purchase and deliver drugs to people who were isolating in hotel rooms so they could remain isolated, without experiencing withdrawal symptoms.

Despite social distancing restrictions, group members emphasized the importance of in-person solutions to the stressors of the dual public-health emergencies. This included small-scale initiatives such as door-to-door grocery delivery to providing medical supplies to those living in informal encampments (also known as ‘tent cities,’), where multiple community members set up informal shelters in a group setting, amidst broader contexts of social-economic deprivation (i.e., unsuitable housing). Outside of essential services, group members also described how they were trying to continue social connections between people who use unregulated drugs. One working group member described a conversation between himself and a friend who, before the pandemic, ran a program called “Monday Munchies” where people cook for each other in their residential buildings, and how they worked together to find a solution that was COVID-safe for everyone—creating a continued sense of community within their buildings during a time of increasing social isolation. Overwhelmingly, group members expressed that workers with lived expertise were in unique positions with the ability and determination to create on-the-ground change that other organizations were failing to accomplish. As one group member said:

*I feel like one of the lessons that I’ve learned and that I hope can be emphasized and taken from all of this is the importance of community-based activity and services. Because with agencies that, and governments and sort of becoming impotent in terms of delivering services to people or engaging people, I think people have had to rely on their own devices, so to speak, on their own creativity and on their own resilience. And being able to build that up moving forward.* (P3, May 2021, Interview)

As the initial pandemic response produced adverse conditions for people who use unregulated drugs, policy-makers eventually began involving people with lived and living expertise into their decision-making processes. This change in the governments’ position was something working group members had described as a necessary catalyst for change in Stage 1 of interviews. As one participant stated:

*I think that’s a big takeaway, that we have a lot of work to do as a society. And I think that* *this—COVID has exposed all of our deficiencies. And I think that it may take policy level* *people to start advocating, like, “Hey, we got to fix this,” right?* (P7, June 2020, Interview).

Throughout the study, working group members regularly emphasized the need for involvement of people with lived and living expertise in policy decisions, with one member in October 2020 proposing an institutional process whereby people with lived and living expertise “vet” potential policies before their implementation:

*Give us a body of people that they vet their policies through, and all of the things that* *they’re doing for people who use drugs, because it’s coming from the top down, and* *they’re just doing a really shitty job. So, I say flip the model on its head and have a body* *of people who use drugs telling them what policies need to be and what’s happening on* *the ground, and allow us to do the outreach with our people and make recommendations* *to them* (P7, October 2020, Roundtable).

Once policymakers began consulting people with lived and living expertise, some working group members expressed frustration with the delayed response and that their voices were only now being heard. However, many saw the uncertainty of the pandemic and the resulting receptivity of federal agencies (such as Health Canada and the Public Health Agency of Canada) to engage them in problem solving as an impetus for positive change. Colloquially, Canadians embraced the ominous phrase, living in “the new normal,” as if social distancing, PPE requirements, virtual work, and indeterminate disruptions to services would last indefinitely. However, this phrase was re-imagined in a unique and hopeful way by some group members who envisioned the pandemic as a potential catalyst for change towards a “better normal,” which encompassed improved circumstances for people who use unregulated drugs via innovative changes and opportunities instigated during this time (e.g., food and medical supply deliveries, better housing access, safe supply). Some members explained that they had no desire to “go back to normal” and are now able to “work towards creating a post-COVID normal” that works for people who use unregulated drugs (July 2021, Roundtable). Another group member affirmed this feeling, saying “We have to make a new—a better place, you know? And hopefully, we’ll do it” (P13, June 2021, Interview)*.* As summarized by this group member:

*I mean, the Government of Canada is saying ‘we’re open to hear, we want to hear from you’ like ‘what do you need?’ and I feel like this is a unique opportunity because it hasn’t been offered like this before. So, I feel like we are at a place where we need to have conversations that can shape how services are delivered, how policies need to change, so that this that we experience can be led by us, can be influenced by us, can be shaped by us, so that we can benefit. It might not be long-lasting because as governments change and different belief systems come in and different ideologies come in, maybe we’ll face kickback and we’ll face hit back, but we have a unique opportunity to figure out how do we shape a post-COVID for people who use drugs.* (P3, June 2021, Roundtable)

## Discussion

At the beginning of the pandemic, public health measures that enforced physical and social distancing resulted in reduced harm reduction service capacity, including reduced hours of operation, relocated service sites, and reduced number of persons allowed in sites [21, 66]. Working group members in this study described these initial disruptions as losing a core sense of ‘purpose’ from helping others. Harm reduction workers with lived experience often have direct connections to community through their social circles, family, loved ones, and those they provide services to, which makes their work more personal; they are never simply supporting clients or patients with whom there is no emotional attachment [49]. The sense of purpose from supporting others—also referred as “compassion satisfaction” [68] among workers in other care professions—has been previously identified as a motivating factor for workers with lived experience continuing their work [34, 51, 59], and as a protective factor against burnout [49]. Losing this core sense of purpose early on in the COVID-19 pandemic impacted the lived experience of working group members in this study, reflecting the importance of their work in promoting, not only the health and wellbeing of others, but also their own.

Some harm reduction services hurriedly transitioned from in-person to virtual service provision [15], which group members described as creating a distinct barrier to providing care to their communities, particularly as their roles rely heavily on interpersonal connection. Other research has reported the difficulties service providers without lived experience (including mental health and primary care providers) recounted in providing equivalent quality of care through virtual methods compared to in-person, given the difficulties in building trusting relationships with clients when working virtually [10, 11, 55]. In line with these findings, working group members in this study who were able to transition from in-person to virtual service provision similarly described feeling disconnected to the people whom they support, which instilled moral distress due to their perceived reduction in quality of care they provided [7]. Moral distress also arose from a perceived sense of “privilege” in working virtually; Working group members working virtually described feeling guilt in regards to their position of relative physical safety from contracting the virus compared to their clients and other harm reduction workers who continued to work in-person.

The repeated loss of their community members to the toxic drug crisis that escalated during the COVID-19 pandemic contributed to the collective suffering, characterised as “cumulative PTSD,” by working group members in this study. Grief felt by workers with lived expertise has been previously described as a unique experience compared to colleagues who do not bring personal/lived expertise to their role [7, 31, 46, 56, 57]. The ‘numbness’ working group members described in this study, has previously been identified as a coping mechanism for workers with lived expertise in order to continue working [56, 57]. Workers with lived expertise who fully embrace their grief have been accused of being “too close to the clients,” and made to feel that this is inappropriate and unprofessional [31], ultimately delegitimizing their sorrow. Ironically, it is these connections between workers with lived experience and service users that are relied upon by harm reduction services to ensure the success of these essential lifesaving programs [44, 72]. While overdose rates soared across the country, working group members in this study expressed a mounting feeling of frustration with the prioritization of public health messaging related to reducing the spread of COVID-19. Working group members described a sense of widespread inattention and lack of concentrated effort to support people who use unregulated drugs in navigating the dual pandemics, which contributed to significant increases in fatal and nonfatal overdoses across Canada. Indeed, previous literature has noted these initial measures, such as reduced harm reduction and social service delivery and closures, as well as enforced physical distancing measures, did impact overdose risk among people who use unregulated drugs [28, 30, 53]. Working group members described this prioritization of one pandemic over the other as an undervaluing of the ongoing toxic drug crisis by government and public health officials, perpetuating a sense that their communities were seen as expendable by these actors and society at large.

Another consequence of the pandemic for workers with lived expertise was a loss of peer support. Working group members described the benefits of talking with their peers about shared traumatic experiences, which has been noted as a key resource utilized by workers in high-trauma fields (e.g., nurses, first responders) to process shared grief and trauma [27, 67, 75]. In addition, peer support for frontline workers has been reported as directly relating to workers’ compassion satisfaction [75]—a major driver of continuing to work in care professions. It important to ensure that workers with lived expertise are able to provide mutually beneficial support for their colleagues, however, it is also imperative that organizations provide better structural supports (e.g., sick pay, paid bereavement leave, access to counseling/therapy) to reduce the responsibilities of individual workers in caring for their colleagues [7]. Our findings emphasize the reliance of workers with lived expertise on the emotional support of their peers, which working group members noted was disrupted during the pandemic. This disruption stemmed from a decrease in in-person interactions and the significant decline in the emotional capacity of workers to support one another, caused by limited institutional supports and the "cumulative PTSD" and grief they experienced.

By late 2020 and into 2021, many services had reopened (with varied and typically reduced capacity) due, in part, to weakened restrictions on physical distancing, yet people who use unregulated drugs continued to contend with the two public health emergencies, and their impacts on one another. Working group members described how harm reduction services faced difficulties keeping pace with the relentless changes in policies, which has been echoed by workers across Canada, who state that the inconsistency and unreliability of harm reduction service provision a year into the pandemic created significant confusion and difficulties reaching those most in need [21, 64]. Meanwhile, the toxic drug crisis did not abate; in 2021 there were 7,873 overdose-related deaths across Canada—an 18.5% increase from 2020 [63].

In response, working group members recounted implementing and joining initiatives in order to support their communities and fill the gaps where organizations and policies were falling short. These rapidly developed, typically short-term, strategies to continue harm reduction work were implemented by workers with lived expertise who were most aware of the needs of the community, even during periods of service and communication disruptions. Workers with lived experience have a long history of implementing and continuing the provision of services, often against the law, and at personal risk [6, 24, 43, 52, 73], as the work needed to support communities requires a deep knowledge of community needs as well as nimble responsiveness to new and ongoing issues [7, 34, 35]. Working group members in this study stressed the importance of in-person solutions, which they were able to accomplish through innovative and lifesaving initiatives—some that could not be implemented by official organizations (e.g., delivering drugs to people who were isolating to mitigate withdrawal symptoms). This commitment highlights workers with lived expertise’s resilience, flexibility, and responsiveness in the face of delayed public health and government interventions. Nonetheless, these responses to crisis should not fall on the hands of marginalized communities, despite a long history of government and public health agencies doing so [37, 39]. The neoliberal shifting of responsibility for care provision to the individual positions workers with lived expertise to the frontlines, while simultaneously perpetuating the socio-structural systems that constrain their ability to make long-term systemic change (e.g., lack of employment benefits, organizational supports and appropriate pay) [7, 56, 56, 57, 57].

Throughout the pandemic, the world began to refer to the dramatic changes in everyday life as a “new normal.” This has been extended to the rise in overdoses during this time, and the resulting complacency and acceptance of this ongoing loss of life by the general population [29]. This concept of “new normal” has been identified in the literature as being distinct for people with lived and living experience, whose socioeconomic and health conditions have worsened overall [40]. However, some working group members in this study reframed this “new normal” as a ‘fresh start,’ where the rapid change required to address the COVID-19 pandemic was viewed as a potential catalyst for rapid organization and public policy changes. Since the outset of the pandemic, more public health and advocacy organizations have come out to advocate for the expectation that people with lived and living experience be identified as experts in the field and necessary consultants [13, 18]. Indeed, the pandemic has been described by others as an opportunity to “re-think, renegotiate and renew our social contract [wherein] we must turn our efforts to righting political wrongs, particularly those perpetrated against the most marginalised.” [18]. Following the disastrous consequences of some initial public health policies and recommendations (e.g., promoting physical distance leading to people using drugs alone), working group members of this study described being ‘invited to the table’ as representatives for their communities and as subject matter experts. As members of a wide range of service organizations and unions of people with lived and living expertise, the working group members are not unfamiliar with consultation opportunities, however, toward the end of our discussions, there was certainly a resonating hope for a “better normal”. While the findings largely align with existing literature, the iterative and collaborative community-based methodological approach reflects this desire for more inclusive approaches, centring the expert perspectives of highly skilled workers with lived expertise as necessary to ensure the findings were in keeping with the rapidly changing landscape of harm reduction service provision during the first year of the COVID-19 pandemic.

At the outset of the People with Lived and Living Expertise of Drug Use National Working Group, recruitment efforts aimed to establish a working group with a diverse range of identities and perspectives. However, there remain a number of limitations in this study. Indigenous voices are absent—a critical gap given that the toxic drug crisis and war on drugs in Canada disproportionately impacts Indigenous peoples due to ongoing colonialism, racism and intergenerational trauma [2, 78]. Additionally, there is potential for geographic bias due to the overrepresentation of group members located in Ontario, British Columbia and Nova Scotia, as well as an overrepresentation of members located in large urban areas (e.g., Vancouver, Edmonton, Toronto, Montreal). This may limit the generalizability of findings to frontline workers with lived expertise of drug use in other provinces and territories. These limitations notwithstanding, this study provides important information on the unique experiences of these workers during the COVID-19 pandemic including, not only the significant challenges to continuing their work structurally and personally, but also workers’ resilience in the face of these challenges.

## Conclusions

This longitudinal study contributes to the existing research on the views of harm reduction workers during the dual health emergencies of COVID-19 and a poisoned drug supply, prioritizing perspectives of workers with lived expertise of substance use. Our findings highlight some of the barriers the working group experienced in supporting others, something that could be addressed through the provision of additional supports for workers with lived experience of drug use such as job security, access to counselling services, sick leave, employment benefits, and through other means such as increased facilitation of peer-to-peer community engagement and cultural support. This is particularly salient after the pandemic exacerbated the social and structural vulnerabilities these workers already faced (e.g., burnout, grief, economic insecurity). This study illustrates the significance of empowering drug-user-led solutions while centering the voices of people with lived expertise in any decision-making that may impact people who use unregulated drugs, during and beyond public health emergencies.

Stories collected in this study revealed many ways in which disruption to harm reduction and other social service provision intensified the impacts of the dual pandemics, causing immense and multifaceted harm to both people who use unregulated drugs and the workers with lived expertise working to support them. Given the ongoing political and media-driven contestation of the provision of harm reduction services (e.g., B.C.’s recent roll-back of take-home prescribed alternatives program, safe supply) [33], and its resulting increase in public stigma toward people who use unregulated drugs, it is critical that evidence-based information is highlighted regarding the necessity of these services and their importance in supporting those most impacted by the toxic drug crisis.

Moving forward, recognizing Canada’s ability to rapidly mobilize, as evidenced by the COVID-19 pandemic, we call for concrete, effective national community-action to address the toxic drug crisis and its drivers (e.g., criminalization; lack of a national housing plan and basic income; on-going colonialism and systemic racism) with people with lived and living expertise at the forefront of all stages of implementation and design.

## Data Availability

The research data included in this paper cannot be shared as it includes sensitive or confidential information regarding the participants of the Working group and, as such, sharing it violate our ethics approval.

## Footnotes

1 Most recent data at time of writing.

2 The authors use the term “expertise” when referring to people with lived and living experience of unregulated drug use that work in harm reduction services and/or drug user-led unions and organizations to recognize their valuable expertise in the field of substance use that they bring to the organizations they work with, including (but not limited to) their lived experience, their locational insight of community needs, and their greater ability to connect with people who access services. However, when referring to specific literature, we use the term “people with lived and living experience” to be consistent with their terminology.
